# Evidence-based treatment for Depersonalisation-derealisation Disorder (DPRD)

**DOI:** 10.1186/2050-7283-1-20

**Published:** 2013-10-28

**Authors:** Eli Somer, Taryn Amos-Williams, Dan J Stein

**Affiliations:** School of Social Work, University of Haifa, Haifa, Israel; Department of Psychiatry, University of Cape Town, Cape Town, South Africa

**Keywords:** Depersonalisation disorder, Derealisation disorder, Depersonalisation-derealisation disorder, Depersonalisation syndrome, Derealisation syndrome, Depersonalisation-derealisation syndrome, Drug, Pharmacotherapy, Medication, Randomised control trial, RCT, Treatment

## Abstract

**Background:**

Depersonalisation-derealisation disorder (DPRD) is a distressing and impairing condition with a pathophysiology that is not well understood. Nevertheless, given the growing interest in its pathogenesis, and the publication of a number of treatment trials, a systematic review of randomised controlled pharmacotherapy and psychotherapy trials is timely.

**Methods:**

A systematic search of articles on DPRD published from January 1980 to August 2012, using Cochrane methods, was conducted. All randomised controlled trials (RCTs) of pharmacotherapy, psychotherapy, somatic interventions and a blend of these modalities for the treatment of depersonalisation disorder were included in the review. Searches were carried out on multiple databases. The bibliographies of all identified trials were checked for additional studies and authors were contacted for published trials. No unpublished trials were found and no restrictions were placed on language and setting. Data extraction sheets were further designed to enter specified data from each trial and risk of bias information was identified. PRISMA guidelines were also followed to ensure that our methodology and reporting were comprehensive. Of the unique 1296 papers that were retrieved, four studies met the inclusion criteria and were reviewed.

**Results:**

Four RCTs (all within the duration of 12 weeks or less) met study criteria and were included (180 participants; age range 18–65 years). The four RCTs included two lamotrigine studies, one fluoxetine study and one biofeedback study. Evidence for the treatment efficacy of lamotrigine was found in one study (Cambridge Dissociation Scale, CDC: p < 0.001) with no evidence of effect for lamotrigine in the second study (CDS: p = 0.61 or Present State Examination: p = 0.17). Fluoxetine and biofeedback were not more efficacious than the control condition, although there was a trend for fluoxetine to demonstrate greater efficacy in those with comorbid anxiety disorder. The four studies had 'low' or 'unclear' risk of bias.

**Conclusion:**

The limited data from randomised controlled trials on the pharmacotherapy and psychotherapy of DPRD demonstrates inconsistent evidence for the efficacy of lamotrigine, and no efficacy for other interventions. Additional research on this disorder is needed.

## Background

Depersonalisation disorder (DPRD), renamed depersonalisation-derealisation disorder in the DSM-5 (Spiegel et al. 2011), is an alteration in the perception or experience of the self and the environment. Individuals with depersonalisation feel uneasily estranged and separated from their selves (depersonalisation) and their surroundings (derealisation), experiencing what was also described as a sense of disembodiment (desomatisation) and a diminution or loss of emotional reactivity (de-affectualisation) (American Psychiatric Association 2000;Medford et al. 2005a;Sierra 2009). Depersonalisation occurs as a persistent, pervasive phenomenon, causing subjective distress and functional impairment (Medford et al. 2005a). Depersonalisation symptoms can occur in many neurological (e.g. migraine and epilepsy, (Lambert et al. 2005)) and psychiatric conditions (e.g. major depression, panic disorder, posttraumatic stress disorder, schizophrenia, stress and fatigue, (Medford 2012)), or it may occur as a primary phenomenon, in which case it is classified as depersonalisation-derealisation disorder (Simeon et al. 1997).

DPRD is frequently a chronic disorder, affecting between 1% and 2.4% of the general population with a gender ratio of about 1:1, although its comorbidity with depression and anxiety falls between the percentage ranges of 20–40 (Bebbington et al. 1997;Hunter et al. 2004a;Ross 1991). Depersonalisation and derealisation symptoms seem to be more common among women (26.5%) than men (19.5%) (Aderibigbe et al. 2001). It was estimated in one survey that DPRD occurred in 80% of psychiatric inpatients and that 12% of them suffered from a severe form of this condition (Brauer et al. 1970). Lifetime prevalence of depersonalisation and derealisation symptoms of 31 and 66% were found in surveys conducted among non-clinical respondents compared to a lifetime prevalence of depersonalisation and derealisation symptoms of 42 to 91% in psychiatric settings (Hunter et al. 2004b). Severe clinical depersonalisation was identified among 1.9% of German participants (Michal et al. 2009) and 5% of psychiatric outpatients in New York (Foote et al. 2006).

Historical reports of the use of barbiturates, amphetamines and antipsychotics in the treatment of DPRD do not suggest any consistent benefit (Ackner 1954;Shorvon 1946). Subsequent single case reports suggest potential efficacy for a wide variety of treatments including benzodiazepines (phenazepam, (Nuller 1982); clonazepam, (Stein & Uhde 1989)), atypical neuroleptic medications (clozapine, (Nuller 1982)), tricyclic anti-depressants (desipramine, (Noyes et al. 1987)), drugs with serotonergic activity (fluoxetine, (Fichtner et al. 1992;Ratliff & Kerski 1995); fluoxetine and buspirone, (Abbas et al. 1995)), SNRIs (venlafaxine, (Preve et al. 2011)), a combination of benzodiazepines and serotonergic activity drugs (citalopram-clonazepam, (Sachdev 2002)), anti-convulsants (lamotrigine, (Sierra et al. 2006)), (methylphenidate, (Foguet et al. 2011)), and opiate antagonists (naltrexone, (Ginsberg 2005)). Other tried psychiatric interventions included electroconvulsive therapy (ECT) (Ordas & Ritchie 1994) and transcranial magnetic stimulation (Jimenez-Genchi 2004). Psychotherapy case reports have indicated that psychodynamic psychotherapy (Torch 1987) and hypnosis-based treatment, combined with eye movement desensitisation and reprocessing (EMDR), (Hollander 2009)), may also be useful.

Several small open-label studies have also been conducted. Based on the hypothesis that emotional numbing is an opiate-mediated phenomenon, nalmefene, an oral opiate antagonist, was administered and reported to lessen depersonalisation symptoms in some combat veterans suffering from PTSD (Glover 1993). Although the duration of the response was not clearly described, a marked decline in chronic depersonalisation was reported in subjects treated intravenously with naloxone, another opiate antagonist (Nuller et al. 2001). In a later open prospective trial of naltrexone administered to 12 participants with DPRD who completed at least four weeks of naltrexone treatment, four (33%) showed marked improvement with a 50% to 90% reduction in symptoms (Simeon & Knutelska 2005).

A different body of research suggests that glutamate might be relevant to dissociation. Sub-anesthetic doses of the N-methyl D-aspartate receptor antagonist ketamine were shown to induce subjective experiences characteristic of depersonalisation (Krystal et al. 1994). It is believed that the altered state of consciousness induced by ketamine is mediated by increased glutamate release in response to NMDA receptor blockades, with a consequent excess of glutamate activity at non-NMDA glutamate receptors (Abel et al. 2003;Pikwar 2011). Lamotrigine has been reported in the treatment of DPRD because of its ability to impede glutamate release at the presynaptic membrane and to reduce the effects of ketamine on consciousness (Anand et al. 2000;Wang et al. 1996). While a crossover, double-blind study on nine patients with DPRD, failed to show any beneficial effects of lamotrigine (Sierra et al. 2003), lamotrigine was reported to benefit some patients with chronic DPRD (Sierra et al. 2006;Sierra et al. 2001) when used as an add-on therapy.

There have also been some publications on psychotherapy research in DPRD. One psychoanalytic case study was mentioned earlier (Torch 1987), and two additional case reports representing behavioral therapy (Sookman & Solyom 1978) and directive therapy (Blue 1979) have been published. However, the last two reports focused on depersonalisation as a co-morbid, secondary disorder. A cognitive–behavioral model of depersonalisation has been developed, and comprises another potential form of treatment. This model is based on evidence that depersonalisation is associated with anxiety rather than with dissociative conditions (Medford et al. 2005b;Hunter et al. 2003;Hunter et al. 2005a).

Nevertheless, the disorder remains a poorly understood condition that has received relatively little research attention. Lack of awareness of DPRD may contribute to a high rate of misdiagnosis (Hunter et al. 2004a). With growing interest in the management of DPRD, it is timely to conduct a systematic review to determine the efficacy of medication, psychotherapy, somatic interventions and a combination of treatment modalities for depersonalisation-derealisation disorder, relative to placebo and other comparison groups.

## Methods

### Identification of studies

The literature search was carried out using the following databases: the Cochrane Central Register of Controlled Trials (CENTRAL) (The Cochrane Library, Issue 8), MEDLINE, PsycINFO, the metaRegister of Controlled Trials database (mRCT), the National Institute of Health's Computer Retrieval of Information on Scientific Projects (CRISP) service, ClinicalTrials.gov, and the WHO International Clinical Trials Registry Platform (ICTRP), for articles published from January 1980 to August 2012. The following search terms (in both American and British English) were used:

“depersonalisation disorder” OR “derealisation disorder” OR “depersonalisation-derealisation disorder” OR “depersonalisation syndrome” OR “derealisation syndrome” OR “depersonalisation-derealisation syndrome” AND “drug” OR “pharmacotherapy” OR “medication” OR “treatment” AND “randomised control trial” OR “RCT”. The initial search yielded 1296 studies, of which four met study criteria and were included. The bibliographies of all identified trials were checked for additional studies and the authors were contacted for published trials. No restriction was placed on language and setting. Studies employing cross-over and parallel designs were potentially considered for inclusion. No unpublished trials were found.

Criteria for considering studies for this review included (a) all randomised controlled trials (RCTs) of pharmacotherapy, psychotherapy, somatic interventions and a combination of treatments for depersonalisation disorder, (b) all participants diagnosed with depersonalisation disorder according to the criteria of the Diagnostic and Statistical Manual (DSM-III-R, (American Psychiatric Association 1987) or DSM-IV, (American Psychiatric Association 1994)), or the International Classification of Diseases (ICD-9, (National Center for Health Statistics 2002) or ICD-10, (World Health Organization 2008)) irrespective of age, in- or outpatient status, or presence of comorbidity, (c) all medication agents and non-pharmacological interventions (e.g. selective serotonin reuptake inhibitors (SSRIs), anticonvulsants and opiate antagonists, temporo-parietal junction stimulation), and (d) RCTs of all forms of psychotherapy (e.g. behavioural modification and cognitive restructuring programs, relaxation, gestalt, interpersonal, supportive therapies, mindfulness, acceptance and commitment therapy, compassion-focused therapy). Both short- and long-term therapy were eligible for inclusion, as was group therapy in which cluster randomisation designs were employed.

Where possible, planned treatment comparisons included:

1. Pharmacotherapy versus placebo.
2. Psychotherapy versus sham interventions or waiting list.
3. Psychotherapy versus pharmacotherapy.
4. Pharmacotherapy versus non-pharmacological interventions.

### Outcome measures and effect variables

#### Primary outcomes

Treatment response was reported if studies used the Clinical Global Impressions-Improvement subscale (CGI-I), a widely used categorical measure of treatment response in which responders are defined as having a change item score of 1 = "very much" or 2 = "much" improved (CGI, (Guy et al. 1976)), or by a 50% reduction reported by the Cambridge Depersonalization Scale (Sierra & Berrios 2000).

The effect of intervention on symptom severity was determined from standardised instruments such as the Cambridge Depersonalisation Scale, the Dissociative Experiences Scale (DES, (Bernstein-Carlson & Putnam 1993)), or the Depersonalisation Severity Scale (DSS, (Simeon et al. 2001)).

### Secondary outcomes

Depression was reported if studies provided data on the 17-item Hamilton Rating Scale for Depression (HRSD, (Hamilton 1960)), the Beck Depression Inventory (BDI, (Beck et al. 1961)), or a similar scale. Anxiety was measured with the standard Hamilton Rating Scale for Anxiety (HRSA, (Hamilton 1959)) the Beck Anxiety Inventory (BAI, (Beck et al. 1988), or a similar scale. Symptom improvement in other anxiety disorders similarly employed customary “gold-standard” severity measures.

### Meta-analysis

The analytical summary of the selected studies was considered but high heterogeneity across studies prohibited combining results to produce a single overall estimate of effect.

### Data collection

#### Selection of studies

In order to determine whether studies were eligible for inclusion, the Cochrane steps of a systematic search were followed. This entailed the screening of titles and abstracts for face validity within the selected databases. Included, excluded and unclear studies were colour coded, and the full text articles for each study were retrieved. After full text screening, studies were further included or excluded based on the study criteria for the review. This process was completed by one of the authors (ES). Spreadsheet forms were designed for the purpose of recording descriptive information, summary statistics of the outcome measures, risk of bias data, and associated commentary (ES and TW). The reviewers contacted investigators by email in an attempt to obtain missing information. A narration of each trial is provided in the results section. PRISMA guidelines (Moher et al. 2009) were also followed to ensure that the methodology and reporting were comprehensive (see Table 1).

**Table 1:** **PRISMA checklist**

Title	#	
Title	1	A systematic review titled: “Evidence-based Treatment for Depersonalisation-derealisation Disorder (DPRD)”.
**Abstract**		
Structured summary	2	*Background*
		Depersonalisation-derealisation disorder (DPRD) is a distressing and impairing condition with a pathophysiology that is not well understood.
		*Objectives*
		A systematic review of randomised controlled pharmacotherapy and psychotherapy trials.
		*Data sources*
		Articles on DPRD published from January 1980 to August 2012. Searches were carried out on The Cochrane Central Register of Controlled Trials (CENTRAL) (The Cochrane Library, Issue 8), MEDLINE, PsycINFO, the metaRegister of Controlled Trials database (mRCT), the National Institute of Health's Computer Retrieval of Information on Scientific Projects (CRISP) service, ClinicalTrials.gov, and the WHO International Clinical Trials Registry Platform (ICTRP).
		*Study eligibility criteria*
		Randomised controlled trials (RCTs).
		*Participants*
		Individuals diagnosed with depersonalisation disorder (i.e. DSM-III-R, DSM-IV, ICD-9 or ICD-10) irrespective of age, in- or outpatient status, or presence of comorbidity.
		*Interventions*
		Pharmacotherapy (e.g. SSRIs), psychotherapy (e.g. behavioural modification and cognitive restructuring programs), somatic interventions (e.g. health education) and a blend of these modalities.
		*Study appraisal methods*
		Data extraction sheets were designed to enter specified data from each trial and risk of bias information was identified.
		*Results*
		Four RCTs (all within the duration of 12 weeks or less) met study criteria and were included (180 participants; age range 18–65 years). The four RCTs included two lamotrigine studies, one fluoxetine study and one biofeedback study. Evidence for the treatment efficacy of lamotrigine was found in one study (Cambridge Dissociation Scale (CDS): p < 0.001) with no evidence of effect for lamotrigine in the second study (CDS: p = 0.61 or Present State Examination (PSE): p = 0.17). Fluoxetine and biofeedback were not more efficacious than the control condition, although there was a trend for fluoxetine to demonstrate greater efficacy in those with comorbid anxiety disorder. The four studies had 'low' or 'unclear' risk of bias.
		*Limitations*
		There are a small number of studies with small samples. There are differences across trials in sample characteristics, and timing of interventions.
		*Conclusion*
		The limited data from randomised controlled trials on the pharmacotherapy and psychotherapy of DPRD demonstrates inconsistent evidence for the efficacy of lamotrigine, and no efficacy for other interventions. Additional research on this disorder is needed.
**Introduction**		
Rationale	3	DPRD is not a rare condition. It occurred in 80% of psychiatric inpatients. A lifetime prevalence of depersonalisation and derealisation symptoms of 42 to 91% was reported in psychiatric settings. Given the growing interest in its pathogenesis, and the publication of a number of treatment trials, a systematic review of randomised controlled pharmacotherapy and psychotherapy trials is timely.
Objectives	4	Lack of awareness of DPRD may contribute to a high rate of misdiagnosis. With growing interest in the management of DPRD, we aimed at conducting a systematic review to determine the efficacy of medication, psychotherapy, somatic interventions and a combination of treatment modalities for depersonalisation-derealisation disorder, relative to placebo and other comparison groups.
**Methods**		
Protocol and registration	5	This systematic search used Cochrane methods (http://www.cochrane.org).
Eligibility criteria	6	RCTs of pharmacotherapy, psychotherapy, somatic interventions and a blend of these modalities for the treatment of DPRD published from January 1980 to August 2012 in any language.
Information sources	7	Searches were carried out on The Cochrane Central Register of Controlled Trials (CENTRAL) (The Cochrane Library, Issue 8), MEDLINE, PsycINFO, the metaRegister of Controlled Trials database (mRCT), the National Institute of Health's Computer Retrieval of Information on Scientific Projects (CRISP) service, ClinicalTrials.gov, and the WHO International Clinical Trials Registry Platform (ICTRP). The bibliographies of all identified trials were checked for additional studies and authors were contacted for published trials.
Search	8	For each database, the following search terms (in both American and British English) were used: “depersonalisation disorder” OR “derealisation disorder” OR “depersonalisation-derealisation disorder”, OR “depersonalisation syndrome” OR “derealisation syndrome” OR “depersonalisation-derealisation syndrome”, AND “drug” OR “pharmacotherapy” OR “medication” OR “treatment” OR “psychotherapy”, AND “randomised control trial”, OR “RCT”. The bibliographies of all identified trials were checked for additional studies and the authors were contacted for published trials. No restriction was placed on language and setting. Studies employing cross-over and parallel designs were potentially considered for inclusion.
Study selection	9	The criteria for selecting studies included (a) all RCTs of pharmacotherapy, psychotherapy, somatic interventions and a combination of treatments for DPRD, (b) all participants diagnosed with DPRD irrespective of age, in- or outpatient status, or presence of comorbidity, (c) all medication agents and non-pharmacological interventions, and (d) RCTs of all forms of psychotherapy. Both short- and long-term therapy were eligible for inclusion, as was group therapy in which cluster randomization designs were employed. Titles and abstracts were screened for face validity within the selected databases. Included, excluded and unclear studies were color coded, and the full text articles for each study were retrieved. After full text screening, studies were further included or excluded based on the study criteria for the review.
Data collection process	10	Study selection was completed by one of the authors (ES). Spreadsheet forms were designed for the purpose of recording descriptive information, summary statistics of the outcome measures, risk of bias data, and associated commentary (ES and TW). The reviewers contacted investigators by email in an attempt to obtain missing information. PRISMA guidelines were also followed to ensure that the methodology and reporting were comprehensive.
Data items	11	Aliyev and Aliyev 2011;Sierra et al. 2003;Simeon et al. 2004 and Schoenberg et al. 2012
		*Participants*
		All participants diagnosed with depersonalisation disorder according to the criteria of the Diagnostic and Statistical Manual (DSM-III-R or DSM-IV), or the International Classification of Diseases (ICD-9 or ICD-10) irrespective of age, in- or outpatient status, or presence of comorbidity.
		*Interventions*
		All medication agents and non-pharmacological interventions (e.g. selective serotonin reuptake inhibitors (SSRIs), anticonvulsants and opiate antagonists, temporo-parietal junction stimulation), and RCTs of all forms of psychotherapy (e.g. behavioural modification and cognitive restructuring programs, relaxation, gestalt, interpersonal, supportive therapies, mindfulness, acceptance and commitment therapy, compassion-focused therapy). Both short- and long-term therapy were eligible for inclusion, as was group therapy in which cluster randomisation designs were employed.
		*Comparisons*
		Where possible, planned treatment comparisons included:
		1. Pharmacotherapy versus placebo.
		2. Psychotherapy versus sham interventions or waiting list.
		3. Psychotherapy versus pharmacotherapy.
		4. Pharmacotherapy versus non-pharmacological interventions.
		*Outcomes*
		Diagnostics & baseline screening: all participants diagnosed with depersonalisation disorder according to the criteria of the Diagnostic and Statistical Manual (DSM-III-R, or DSM-IV), or the International Classification of Diseases (ICD-9, or ICD-10).
		Primary measures: Treatment response was reported if studies used the Clinical Global Impressions-Improvement subscale (CGI-I), a widely used categorical measure of treatment response in which responders are defined as having a change item score of 1 = "very much" or 2 = "much" improved (CGI), or by a 50% reduction reported by the Cambridge Depersonalization Scale. The effect of intervention on symptom severity was determined from standardised instruments such as the Cambridge Depersonalisation Scale, the Dissociative Experiences Scale (DES), or the Depersonalisation Severity Scale (DSS).
		Secondary measures: Depression was reported if studies provided data on the 17-item Hamilton Rating Scale for Depression (HRSD), the Beck Depression Inventory (BDI), or a similar scale. Anxiety was measured with the standard Hamilton Rating Scale for Anxiety (HRSA) the Beck Anxiety Inventory (BAI, or a similar scale. Symptom improvement in other anxiety disorders similarly employed customary “gold-standard” severity measures.
Risk of bias in individual studies	12	The overall risk of bias was evaluated as 'high’, 'low’ or 'unclear’ according to the five criteria stipulated by the Cochrane Handbook for Systematic Reviews of Interventions: random sequence generation, allocation concealment, blinding (performance bias and detection bias), blinding of outcome assessment, incomplete outcome data (attrition bias), and selective reporting (reporting bias).
Summary measures	13	Treatment response was reported if studies used the Clinical Global Impressions-Improvement subscale (CGI-I), a measure of treatment response in which responders are defined as having a change item score of 1 = "very much" or 2 = "much" improved (CGI), or by a 50% reduction reported by the Cambridge Depersonalization Scale (CDS). The effect of intervention on symptom severity was determined from standardized instruments such as the CDS, the Dissociative Experiences Scale (DES), or the Depersonalization Severity Scale.
Synthesis of results	14	Due to the clinically diverse nature of each trial, with different interventions used in different studies, the trials could not be meta-analysed.
Risk of bias across studies	15	All four studies were rated as having an “unclear” risk of bias for selective reporting, because there was no protocol available to determine if all outcomes were measured.
Additional analyses	16	N/A.
**Results**		
Study selection	17	Records identified through database searching: n = 1296; Records excluded: n = 237, Reason: Duplicates; Title screening: n = 1059; Records excluded: n = 341, Reason: Not Depersonalisation/derealisation; Abstract screening: n = 718; Records excluded: n = 689, Reason: Not treatment articles or no outcome provided; Full-text articles assessed for eligibility: n = 29; Full-text articles excluded: n = 25, Reasons: Retrospective studies and open trials; Studies included in qualitative synthesis: n = 4.
Study characteristics	18	Medication
		● Aiyev 2011: Azerbaijani outpatients, single center, lamotrigine (25–300 mg/day), placebo comparison, 12 weeks, 80 randomised, mean age 37.7; 0% female, diagnostics: CDS.
		● Sierra et al. 2003: UK outpatients, single center, Lamotrigine (25–250 mg/day), placebo comparison, 12 weeks, 14 randomised, mean age 35.2, diagnostics: DSM-IV, PSE, CDS.
		● Simeon 2004: USA outpatients, single center, Fluoxetine (10–60 mg/day Eli Lilly); placebo comparison, 10 weeks, 54 randomised, mean age 36, 39% female, diagnostics: SCID-D.
		Psychotherapy
		● Schoenberg et al. 2012: UK outpatients, single center, electro-dermal biofeedback, sham electro-dermal biofeedback, 4 weeks (8 sessions), 32 randomised, mean age 35, 25% female, diagnostics: SCID-D.
Risk of bias within studies	19	Medication
		● Aliyev and Aliyev 2011: random sequence generation - low; allocation concealment - low; blinding (performance bias and detection bias) - low; blinding of outcome assessment - low; incomplete outcome data - low; selective outcome reporting - unclear.
		● Sierra et al. 2003: random sequence generation - low; allocation concealment - low; blinding (performance bias and detection bias) - unclear; blinding of outcome assessment - low; incomplete outcome data - low; selective outcome reporting - unclear.
		● Simeon et al. 2004: random sequence generation - low; allocation concealment - low; blinding (performance bias and detection bias) - unclear; blinding of outcome assessment - low; incomplete outcome data - low; selective outcome reporting - unclear.
		Psychotherapy
		● Schoenberg et al. 2012: random sequence generation - unclear; allocation concealment - low; blinding (performance bias and detection bias) - low; blinding of outcome assessment - low; incomplete outcome data - low; selective outcome reporting - unclear.
Results of individual studies	20	Medication
		● Simeon et al. 2004: Fluoxetine was not superior to placebo except for a clinically minimal but statistically significantly greater improvement in CGI–I score in the fluoxetine group. In participants with a comorbid diagnosis of depressive or anxiety disorder, those taking fluoxetine consistently tended to have better responses than those taking the placebo.
		● Sierra et al. 2003: A cross-over study among nine individuals suffering from DPRD comparing the lamotrigine with a placebo revealed following a 2-week washout that lamotrigine had no significant advantage over placebo when administered singularly for DPRD.
		● Aliyev and Aliyev 2011: 12 weeks of lamotrigine therapy resulted in a statistically significant difference in improvement in a lamotrigine group compared with that in the placebo group.
		Psychotherapy
		● Schoenberg 2012: While electro-dermal biofeedback did not help DPRD participants increase skin conductance response, real-time biofeedback resulted in lower state (but not trait) scores on the CDS. Biofeedback had no effect on DES, BDI or BAI scores, compared to sham biofeedback.
Synthesis of results	21	N/A
Risk of bias across results	22	There was no protocol available to determine if all outcomes were measured in the four selected studies. Risk of bias for selective reporting were rated as “unclear” for all included studies.
Additional analyses	23	N/A
**Discussion**		
Summary of evidence	24	Data on lamotrigine for DPRD was inconsistent with one trial indicating that lamotrigine was not significantly better than placebo when applied as a singular treatment for DPRD, and one trial showing a statistically significant difference in improvement compared placebo. Fluoxetine was not demonstrated to be efficacious in treating depersonalization disorder. However, there was a tendency for depersonalization symptoms to improve in subjects with a comorbid anxiety disorder. Electro-dermal biofeedback was not effective in increasing SCR (a marker of emotional response) or in decreasing trait measures of depersonalization. However, SCR biofeedback did result in lower state scores on the CDS.
Limitations	25	We identified a small number of studies with small samples. There are differences across trials in sample characteristics and timing of interventions. Although we used a rigorous search methodology, we may have missed unpublished trials; there is, for example, a bias against the publication of negative studies.
Conclusions	26	There is inconsistent evidence to support the efficacy of lamotrigine in DPRD, with no evidence to support the efficacy of fluoxetine and biofeedback. Further research is necessary, particularly in light of the methodological differences between studies.
**Funding**		
Funding	27	No funding was available for this review.

## Results

### Results of the search

**Figure 1:**
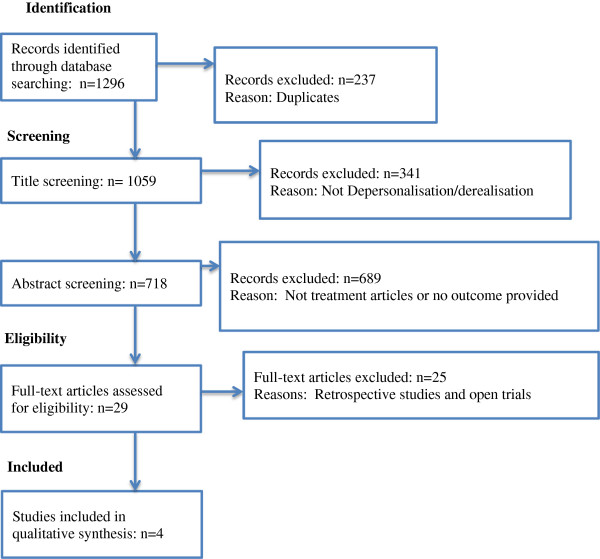
**Flow of information through the different phases of the systemic review.**

MEDLINE, ClinicalTrials.gov, WHO trials and PsycINFO searches retrieved 1147 and 149 unique articles, respectively. The search of the CCDAN Controlled Trials Registry yielded two additional results. Reviews of reference lists of key studies identified one more study, resulting in a total of 1296 unique abstracts (see Figure 1). Of the 14 open and cross-over trials, one was a double-blind, placebo-controlled study that was selected for this review. Four double-blind, placebo-controlled studies (three randomised and one cross-over) were finally selected for independent assessment by two raters (ES and DS). No unpublished trials were found.

### Description of included studies

The search included four double-blind RCTs of treatment for depersonalisation (180 participants, see Table 2). A placebo comparison group was employed in each study and the four studies consisted of one psychotherapy (biofeedback) and three pharmacotherapy trials (two lamotrigine and one fluoxetine). Each study was published in English and recruited outpatients from single centres. One trial was funded by the National Institute of Mental Health (NIMH) (the fluoxetine and placebo capsules were provided by Eli Lilly) and another by the Medical Research Council (MRC) (Schoenberg et al. 2012;Simeon et al. 2004). Countries in which studies were conducted included the United Kingdom (Schoenberg et al. 2012;Sierra et al. 2003), the United States of America (Simeon et al. 2004) and Azerbaijan (Aliyev & Aliyev 2011).

**Table 2:** **Randomized controlled trials included in the review (n = 4)**

Study ID	Funding	Country & Setting	Intervention	Comparison	Weeks (sessions)	Total randomised	Mean age of sample	% female in sample	Diagnostics & baseline screening	Primary measures	Secondary measures	Drop-outs	Lost to follow-up
**Medication**													
Aliyev and Aliyev 2011	None	Azerbaijan outpatients (single centre)	Lamotrigine (dose: 25–300 mg/day)	Placebo	12	80	37.7	0	CDS	CDS	Improvement	15	-
Sierra et al. 2003	None	UK outpatients (single centre)	Lamotrigine (dose: 25–250 mg/day)	Placebo	12	14	35.2		DSM-IV	PSE	DES	5	-
									PSE	CDS	BDI		
									CDS				
Simeon et al. 2004	NIMH grant	USA outpatients (single centre)	Fluoxetine (dose: 10–60 mg/day) (provided by Eli Lilly)	Placebo	10	54	36	39	SCID-D	CGI-I	HRSA	13	-
									Semi-structured clinical interview	DES-DP	HRSD		
										DSS	LSAS		
											YBOCS		
											Panic attacks		
**Psychotherapy**													
Schoenberg et al. 2012	Medical Research Council	UK outpatient (single centre)	Electro-dermal biofeedback (8 sessions)	Sham electro-dermal biofeedback	4 (8)	32	35	25	SCID-D	CDS	-	2	-
										DES			
										BAI			
	Pilkington Pilozzo Charitable Trust									BDI			

The average sample size was 44 and ranged from 14 (Sierra et al. 2003) to 80 (Aliyev & Aliyev 2011). Three studies consisted of both males and females, and one study males only (Aliyev & Aliyev 2011) (mean age for all four groups: 36 years). Amongst others, common inclusions were: adults aged 18–65 years; DSM–IV or PSE (Sierra et al. 2003) diagnostic criteria for current depersonalisation disorder; and written informed consent. Participants were excluded if they had a lifetime diagnosis of schizophrenia, schizoaffective disorder, bipolar disorder, organic mental disorder and substance use disorder (Sierra et al. 2003;Schoenberg et al. 2012), eating disorder, acute or unstable medical illnesses (Simeon et al. 2004), as well as those with a history of seizure disorder or major head trauma. Pregnant and lactating women were also excluded (Schoenberg et al. 2012).

The duration of treatment for all interventions ranged between eight sessions of psychotherapy (Schoenberg et al. 2012) to 12 weeks of pharmacotherapy (Sierra et al. 2003;Aliyev & Aliyev 2011). Dose of medication in the pharmacological studies ranged from 10 mg/day (Schoenberg et al. 2012) to 300 mg/day (Simeon et al. 2004). Primary and secondary outcomes include CDS, DES (Sierra et al. 2003;Schoenberg et al. 2012;Aliyev & Aliyev 2011), BAI (Schoenberg et al. 2012) and BDI (Sierra et al. 2003;Schoenberg et al. 2012), PES, CGI-I, DSS, HRDS, HRSA, LSAS, YBOCS and DSM-IV (Sierra et al. 2003). Two participants (one female and one male) dropped out of the sham condition in the biofeedback study. No side effects were reported in this study (Schoenberg et al. 2012). Fifteen dropouts were reported (three because of the development of a rash in the medication group (Aliyev & Aliyev 2011) compared to five dropouts (Sierra et al. 2003) in two studies investigating lamotrigine). Seven participants further dropped out due to various side effects (i.e. dizziness, muscle aches, nausea, sedation, fatigue and neutropenia) in these two studies (Sierra et al. 2003). In addition, thirteen participants dropped out of the fluoxetine study. Side effects in at least 10% of the two study groups were reported in this trial (Simeon et al. 2004).

### Description of excluded studies

Sixteen abstracts concerning case and retrospective studies on the treatment of DPRD were identified but excluded from this study due to inadequate sample size and lack of a control group. Described interventions included SSRIs (Hollander et al. 1990), benzodiazepines and anti-psychotics (Nuller 1982), an opioid receptor antagonist (Glover 1993), lamotrigine as a single (Sierra et al. 2001) or add-on treatment (Sierra et al. 2006), the opioid receptor antagonists naloxone (Simeon & Knutelska 2005) and naltrexone (Ginsberg 2005;Simeon & Knutelska 2005). One open label trial on temporo-parietal junction stimulation (Mantovani et al. 2011) and cognitive-behavior therapy (Hunter et al. 2005b) was also excluded.

### Risk of bias within studies

The overall risk of bias was evaluated as 'high’, 'low’ or 'unclear’ according to the five criteria stipulated by the Cochrane Handbook for Systematic Reviews of Interventions (Higgins & Green 2008): random sequence generation, allocation concealment, blinding (performance bias and detection bias), blinding of outcome assessment, incomplete outcome data (attrition bias), and selective reporting (reporting bias) (see Table 3).

**Table 3:** **Risk of bias in selected studies**

Study ID	1	2	3	4	5	6
**Pharmacotherapy**						
Aliyev and Aliyev 2011	Low	Low	Low	Low	Low	Unclear
Sierra et al. 2003	Low	Low	Unclear	Low	Low	Unclear
Simeon et al. 2004	Low	Low	Unclear	Low	Low	Unclear
**Psychotherapy**						
Schoenberg et al. 2012	Unclear	Low	Low	Low	Low	Unclear

1: random sequence generation; 2: allocation concealment; 3: blinding (performance bias and detection bias); 4: blinding of outcome assessment; 5: incomplete outcome data; 6: selective outcome reporting.

#### Random sequence generation

Three trials were rated as having a “low” risk of bias on the basis of random sequence generation (i.e. use of a randomisation table, list or code) (Sierra et al. 2003;Simeon et al. 2004;Aliyev & Aliyev 2011). One trial was rated “unclear” (Schoenberg et al. 2012), as the trial indicated that the participants were randomised however the procedure was not clearly defined.

#### Allocation concealment

All four studies were rated as having a 'low’ risk of bias on the basis of allocation concealment. The pharmacological studies all used identical appearing capsules for the medication and placebo groups (Sierra et al. 2003;Simeon et al. 2004;Aliyev & Aliyev 2011). For the psychotherapy study, biofeedback and sham was presented on an identical interface (Schoenberg et al. 2012).

#### Blinding of participants, assessors and personnel

Two trials were rated as having a 'low’ risk for performance and detection bias. In the first trial both the patient and the treating psychiatrist were blinded to treatment (Aliyev & Aliyev 2011), whereas the second study was patient blind (Schoenberg et al. 2012). The additional two trials were rated 'unclear’ (Sierra et al. 2003;Simeon et al. 2004), as they did not provide evidence to determine if blinding occurred.

#### Blinding of outcome assessment

Each trial was rated as having a “low” risk of bias on the basis of blinding of the outcome assessment. For the biofeedback group procedures were identical to those in experimental group (Schoenberg et al. 2012). There was no clear need for the outcome assessments to be blind in the additional three trials (Sierra et al. 2003;Simeon et al. 2004;Aliyev & Aliyev 2011).

#### Incomplete outcome data (attrition bias)

All four studies were rated “low” for attrition bias because all outcomes were reported on. For two studies, dropouts were excluded from the analysis, with no reasons given for dropouts (Sierra et al. 2003;Aliyev & Aliyev 2011). No baseline scores were reported on in one study using lamotrigine (Aliyev & Aliyev 2011).

#### Selective reporting (reporting bias)

For selective reporting, all four studies were rated as having an “unclear” risk of bias, because there was no protocol available to determine if all outcomes were measured.

#### Other biases

All four studies were posed with several limitations. In the biofeedback study (Schoenberg et al. 2012), half the patients (17 participants) were on various medications which may have affected autonomic response; however, the two conditions were similar in terms of the ratio between medicated and non-medicated patients, and medication status was not a significant confound. There was a preponderance of men in the DPRD group compared to the control group, but significant between-group effects were still evident after covarying for age and sex. Only thirty two participants were randomised, this is small in comparison to other clinical trials. Finally, the experimenter was not blind to patient allocation; it is theoretically possible that results were affected by indirectly expressed indicants as to the treatment condition. This study was also implemented over four weeks which may have been short for evidence of an effect.

The two studies investigating lamotrigine also had important limitations. The first study (Aliyev & Aliyev 2011) consisted of eighty men, but no female participants, so preventing generalisation of results to women. Some patients were also allowed to take clonazepam for insomnia and hydroxyzine for the treatment of a rash in concurrent with lamotrigine. Furthermore, the study only reported on those participants who completed the study. The second study (Sierra et al. 2003) used both males and females in their analysis, but only fourteen participants were randomised over twelve weeks, and dropout rates were high.

In the SSRI study (Simeon et al. 2004), fifty-four patients were treated over ten weeks with 10-60 mg of fluoxetine or matching placebo. Some participants were treated with psychotherapy (e.g. cognitive behavioral therapy) for three months, and were nevertheless included in the analysis. Intention-to-treat analysis was used, with last observation carried forward for those participants who did not complete the study. This study was also characterised by high withdrawal rates. Well-validated measures were used and the independent evaluator was masked to side effects and medication adjustment.

### Effects of interventions

#### Pharmacotherapy versus placebo

Fluoxetine (dose 10-60 mg/day) was not superior to placebo on three primary outcome measures, except for a clinically minimal but statistically significantly greater improvement in CGI–I score in the fluoxetine group (2.9 vs. 3.6) (Simeon et al. 2004). In participants with a comorbid diagnosis of depressive or anxiety disorder, those taking fluoxetine consistently tended to have better responses than those taking the placebo (Simeon et al. 2004).

A 12-week double-blind, placebo-controlled, cross-over study among nine individuals suffering from DPRD, comparing the anticonvulsive lamotrigine (dose 25-250 mg/day) (Sierra et al. 2003) with a placebo, revealed following a 2-week washout that lamotrigine had no significant advantage over placebo when administered singularly for DPRD as none of the participants was identified as a responder to the lamotrigine arm of the cross-over. Subsequently, 12 weeks of lamotrigine therapy (25–300 mg/day) resulted in a statistically significant difference in improvement (defined as a 50% reduction in the Cambridge Depersonalisation Scale) in the lamotrigine group compared with that in the placebo group ((Aliyev & Aliyev 2011); ×2 = 22.68, df = 1, p < 0.001).

### Psychotherapy versus sham psychotherapy

While electrodermal biofeedback did not help DPRD participants increase skin conductance response (an hypothesised index of emotional responsiveness), real-time biofeedback resulted in lower state (but not trait) scores on the Cambridge Depersonalisation Scale [early vs. late mean scores and standard deviations in the real-time biofeedback group: 36.0 (16.9) vs. 29.9 (18.9), p = 0.01; compared to 30.5 (14.7) vs. 31.8 (14.9), p = 0.63], scores obtained from patients exposed to sham biofeedback. Biofeedback had no effect on DES, BDI or BAI scores, compared to sham biofeedback.

#### Meta-analysis

Due to the clinically diverse nature of each trial, with different interventions used in different studies, the trials could not be meta-analysed.

## Discussion

To the best of our knowledge, this is the first systematic literature review on the treatment of depersonalisation-derealisation disorder. Four RCTs (all within the duration of 12 weeks or less) were found and included in the study (180 participants; age range 18–65 years). These four RCTs included one psychotherapy (i.e. biofeedback) and three pharmacotherapy (i.e. two lamotrigine and two fluoxetine) trials, with comparison groups.

Data on lamotrigine for DPRD was inconsistent with one trial indicating that lamotrigine was not significantly better than placebo when applied as a singular treatment for DPRD, and one trial showing a statistically significant difference in improvement (i.e., 50% reduction in the CDS) compared placebo (Aliyev & Aliyev 2011). Fluoxetine was not demonstrated to be efficacious in treating depersonalisation disorder. However, there was a tendency for depersonalisation symptoms to improve in subjects with a comorbid anxiety disorder (Simeon et al. 2004). Finally, electrodermal biofeedback was not effective in increasing SCR (a physiological marker of emotional response) or in decreasing trait measures of depersonalization (CDS). However, SCR biofeedback did result in lower state scores on the CDS.

The RCTs included here demonstrated 'low' or 'unclear' risk of bias. Three studies provided evidence for random generation sequence (Sierra et al. 2003;Simeon et al. 2004;Aliyev & Aliyev 2011), four for allocation concealment, two for blinding (Schoenberg et al. 2012;Aliyev & Aliyev 2011), and all four for incomplete outcome data; consistent with ratings of a “low” risk of bias. All four studies had missing study protocols so selective reporting could not be assessed; this is consistent with an 'unclear' risk of bias (see Table 3).

The literature has shown that depersonalisation symptoms can be induced by serotonin receptor agonists such as meta-chlorophenylpiperazine (Simeon et al. 1995), and by substances which act as serotonin agonists such as cannabis (Shorvon 1946), lysergic acid diethylamide, and “ecstasy” (McGuire et al. 1994). Serotonin reuptake inhibitors (SSRIs) were reported to be associated with positive treatment outcome in eight individuals with DPRD and comorbid obsessive-compulsive and panic disorders in a case series (McGuire et al. 1994). Furthermore, in a double-blind crossover trial consisting of eight weeks of desipramine and eight weeks of clomipramine, there was limited evidence that clomipramine was more efficacious than desipramine. Nevertheless, in the only randomized controlled trial of a SSRI in DPRD, fluoxetine was not found efficacious.

It is important to recognise the limitations of the existing literature. There are a small number of studies, each of which has a relatively small sample size. In addition, there are differences across trials in sample characteristics, and duration of the interventions. Finally, although we used a rigorous search methodology, we may have missed unpublished trials; there is, for example, a bias against the publication of negative studies.

Given the limited data available, there is arguably a need for additional research on lamotrigine, other anticonvulsants, SSRIs, opiate antagonists, and repetitive transcranial magnetic stimulation (rTMS).

## Conclusion

There is inconsistent evidence to support the efficacy of lamotrigine in DPRD, with no evidence to support the efficacy of fluoxetine and biofeedback. Given the limited data available, further exploration of lamotrigine, other anticonvulsants, SSRIs, opiate antagonists, and repetitive transcranial magnetic stimulation (rTMS) in larger trials may be useful. Indeed, a great deal of further research on the pathogenesis and treatment of depersonalisation-derealisation disorder is required.
